# The Impact of COVID-19–Related Restrictions on Social and Daily Activities of Parents, People With Disabilities, and Older Adults: Protocol for a Longitudinal, Mixed Methods Study

**DOI:** 10.2196/28337

**Published:** 2021-09-01

**Authors:** Holly Reid, William Cameron Miller, Elham Esfandiari, Somayyeh Mohammadi, Isabelle Rash, Gordon Tao, Ethan Simpson, Kai Leong, Parmeet Matharu, Brodie Sakakibara, Julia Schmidt, Tal Jarus, Susan Forwell, Jaimie Borisoff, Catherine Backman, Adam Alic, Emily Brooks, Janice Chan, Elliott Flockhart, Jessica Irish, Chihori Tsukura, Nicole Di Spirito, William Ben Mortenson

**Affiliations:** 1 Department of Occupational Science and Occupational Therapy Faculty of Medicine University of British Columbia Vancouver, BC Canada; 2 Rehabilitation Research Program GF Strong Rehabilitation Centre Vancouver, BC Canada; 3 International Collaboration on Repair Discoveries Vancouver, BC Canada; 4 Graduate Program in Rehabilitation Sciences Faculty of Medicine University of British Columbia Vancouver, BC Canada; 5 Chronic Disease Prevention Program Southern Medical Program University of British Columbia Vancouver, BC Canada; 6 Rehabilitation Engineering Design British Columbia Institute of Technology Burnaby, BC Canada

**Keywords:** COVID-19, longitudinal study, spinal cord injury, disability, adult, occupational disruption, stroke, older adults

## Abstract

**Background:**

The COVID-19 pandemic has led to wide-scale changes in societal organization. This has dramatically altered people’s daily activities, especially among families with young children, those living with disabilities such as spinal cord injury (SCI), those who have experienced a stroke, and older adults.

**Objective:**

We aim to (1) investigate how COVID-19 restrictions influence daily activities, (2) track the psychosocial effects of these restrictions over time, and (3) identify strategies to mitigate the potential negative effects of these restrictions.

**Methods:**

This is a longitudinal, concurrent, mixed methods study being conducted in British Columbia (BC), Canada. Data collection occurred at four time points, between April 2020 and February 2021. The first three data collection time points occurred within phases 1 to 3 of the Province of BC’s Restart Plan. The final data collection coincided with the initial distribution of the COVID-19 vaccines. At each time point, data regarding participants’ sociodemographics, depressive and anxiety symptoms, resilience, boredom, social support, instrumental activities of daily living, and social media and technology use were collected in an online survey. These data supplemented qualitative videoconference interviews exploring participants’ COVID-19–related experiences. Participants were also asked to upload photos representing their experience during the restriction period, which facilitated discussion during the final interview. Five groups of participants were recruited: (1) families with children under the age of 18 years, (2) adults with an SCI, (3) adults who experienced a stroke, (4) adults with other types of disabilities, and (5) older adults (>64 years of age) with no self-reported disability. The number of participants we could recruit from each group was limited, which may impact the validity of some subgroup analyses.

**Results:**

This study was approved by the University of British Columbia Behavioural Research Ethics Board (Approval No. H20-01109) on April 17, 2020. A total of 81 participants were enrolled in this study and data are being analyzed. Data analyses are expected to be completed in fall 2021; submission of multiple papers for publication is expected by winter 2021.

**Conclusions:**

Findings from our study will inform the development and recommendations of a new resource guide for the post–COVID-19 period and for future public health emergencies.

**International Registered Report Identifier (IRRID):**

DERR1-10.2196/28337

## Introduction

COVID-19 is the third zoonotic virus to infect humans in as many decades and was first identified in Wuhan, China, near the end of 2019 [1]. The origin of the virus, course of transmission, and treatment of infection are still under investigation, but initial understandings of the epidemiology suggest a genome that is 75% to 80% identical to SARS-CoV [1]. Once the severity of the virus was realized, the World Health Organization characterized COVID-19 as a pandemic on March 11, 2020 [2], and recommended the implementation of an evolving series of public health and social measures. These included “measures or actions by individuals, institutions, communities, local and national governments and international bodies to slow or stop the spread of COVID-19” [3]. At a community level these measures involve employees working from home where possible, distance learning, avoiding crowding, wearing a mask, closure of nonessential services, reorganization of health care services, and government-directed calls to stay at home. For the purpose of this paper, the term “restrictions” will be used to refer to the abovementioned measures, including physical distancing and limited in-person interactions with others. The first COVID-19 case in Canada was reported on January 25, 2020 [4]. The initial response by the Canadian government included calls for social distancing, which was later clarified to mean keeping physical distance from others [4], although these terms continue to be used interchangeably in many contexts. As health is a provincial responsibility in Canada, the restrictions vary by province and are subject to change depending on the number of COVID-19 cases in the given location.

Concerns have been raised about the unintended negative consequences of the pandemic restrictions among a variety of groups, including families with young children, those living with disabilities such as spinal cord injury (SCI) or stroke, and older adults. British Columbia’s (BC’s) Restart Plan [5] consists of four phases calling for varying restrictions primarily related to physical distancing (Table 1), resulting in decreased in-person interactions and disruption of social and daily activities.

**Table 1:** British Columbia’s Restart Plan for COVID-19 restrictions.

Phase start date	Restriction period	Restrictions
March 2020	Phase 1	Essential services reopen or remain open in compliance with provincial health orders Declaration of public health emergency Banned mass gatherings of >50 people Restricted visitation in health care facilities
May 18, 2020	Phase 2	Many businesses to reopen with extra precautions and physical distancing measures in place Childcare, hairdressers, gyms, salons, and other services reopen Medical services, such as psychology, dentistry, massage, chiropractic, and occupational and physical therapies, resume
June 24, 2020	Phase 3	Faith-based organizations resume in-person gatherings up to 50 people Designated visitors limited to one person for those living in long-term or assisted-living care facilities Limited hours of operation for restaurants, bars, cafés, and breweries with distancing measures in place
Conditional	Phase 4	Entering this phase is dependent on community immunity, wide vaccination, and broad successful treatments Once one of the three above factors is met, larger gatherings at concerts, conventions, tourism events, and other events can resume

Easing of restrictions to allow transition between phases is dependent on new developing knowledge about COVID-19, tracking of confirmed and recovered cases, new outbreaks, and observing how other regions are responding to the pandemic [5]. The restrictions, in place to reduce transmission and ultimately stop the spread of the virus [3], have many unintended consequences, such as financial strain along with psychosocial implications. In an attempt to mitigate the financial stressors that many people are experiencing, the BC Recovery Benefit was offered as “a one-time direct deposit payment for eligible families, single parents or individuals” [6]. The Canadian Emergency Response Benefit was an additional income support measure that eligible Canadians could claim [7]. These programs offer support for lost income resulting from job loss and ongoing unemployment due to the COVID-19 pandemic; however, they do not address the psychosocial issues that arise with the loss of employment, changes in routine, and overall disruption to social and daily activities, which are vital determinants of health [8]. The loss in daily routine combined with physical distancing can lead to increased isolation and loneliness, ultimately reducing mental health and overall well-being [8]. It is important to consider how these psychosocial changes may occur for families with young children, those living with disabilities such as SCI and stroke, and older adults.

For parents with school-aged children, COVID-19 responses have led to school closures and home-based online learning, which has resulted in an increased need for parental supervision [9]. This has placed additional stressors on parents, who may be dealing with employment changes (eg, unemployment, reduced hours, and working from home) and financial pressures; they also must respond to changing needs for their children’s schooling and daycare. Further challenges may arise for single-parent families, those who have restricted access to out-of-household familial supports, and those experiencing domestic violence [10]. Early COVID-19 studies indicated that negative mood increased for parents and children and “work disruptions” also increased [11]. These findings suggest that parents and their children are facing unique challenges during the COVID-19 pandemic that are impacting their mood and productivity and are likely contributing to declining mental health for the family as a whole [11].

The risks associated with COVID-19 may be further exacerbated in vulnerable populations, such as individuals with disabilities who may have compromised immune systems or face mobility challenges. According to the Canadian Survey on Disability, 81.3% of people with disabilities report using an aid or assistive device, such as a wheelchair or hand and arm supports, to facilitate movement [12]. Using mobility aids or equipment presents additional risks specific to COVID-19, as some prevention strategies may be more difficult for people with disabilities [3]. For instance, they may not be able to stay 1 to 2 meters away from others if they rely on a caregiver for personal care, or they may not be able to don a mask independently [13].

Additionally, it is important to consider the concerns regarding the reduced well-being among older adults due to physical distancing and isolation resulting from these COVID-19 restrictions [14]. Due to the increased risk of infection and fatality among older adults, aged 65 years and over, health officials advise this group “to stay home, and self-isolate” [15]. This has resulted in considerable changes in the daily and social activities for the 8 million older Canadians [5]. Furthermore, there are already indications that the well-being of older adults has declined due to physical distancing and isolation resulting from COVID-19 restrictions [14,16].

At this time, there is limited understanding of how prolonged restrictions influence social and daily activities as well as health and well-being, particularly among families with children, people with disabilities such as SCI or stroke, and older adults. It is crucial to consider the unique challenges that COVID-19 restrictions create for those who may not have been considered when these policies were developed [2,5]. Having more inclusive guidance requires a deeper level of understanding of the lived experience of various populations during the shifting and wide-reaching pandemic restriction period. Therefore, the aims of this study are (1) to understand how COVID-19 restrictions change what people do and how they carry out their daily activities, (2) to track the psychosocial effects of these restrictions over time, and (3) to identify strategies to mitigate the negative potential effects of these restrictions.

## Methods

### Overview

This study used a longitudinal, concurrent, mixed methods design with four data collection time points: time point 1 (T1), time point 2 (T2), time point 3 (T3), and time point 4 (T4). Qualitative description was used as the qualitative methodology for this study [17-19]. The Good Reporting of A Mixed Methods Study (GRAMMS) guideline [20] has been followed for the study protocol and for reporting the results. Quantitative data were collected using an online survey with self-reported questionnaires that participants completed 7 days prior to each interview.

### Eligibility Criteria

Community-dwelling adults over the age of 19 years living in BC, who self-identified as being comfortable writing and speaking in English, were recruited. Participants were excluded if they reported having moderate to severe cognitive impairment or aphasia.

A heterogeneous sample of 81 adults in BC were recruited. This included (1) parents with school-aged children (10/81, 12%), (2) people with SCI (22/81, 27%), (3) people who have experienced a stroke (26/81, 32%), (4) individuals with other disabilities (13/81, 16%), and (5) older adults without self-identified disabilities (10/81, 12%).

### Recruitment and Consent

Participants were recruited using the following methods: (1) online postings at the International Collaboration on Repair Discovery and Reach BC websites, (2) reaching out to people who have consented to being contacted again from previous studies, and (3) advertisements on the researchers’ social media pages (eg, Twitter and Facebook). Recruitment took place during the initial phase of restrictions in BC, from April to May 2020, and the number of participants was ultimately limited by funding. Those interested in participating contacted the research team by phone or email to learn more about the study and determine eligibility. Informed consent was obtained at each study time point.

### Patient and Public Involvement

To date, patients or the public were not involved in the design, conduct, reporting, or dissemination plans of our research. However, this is a protocol paper, and the research is not complete. Patients or the public will likely still be included in the dissemination plans of our research, as the potential toolkit to be developed from this research will involve input from participants.

### Data Collection and Outcome Measures

#### Overview

We used Qualtrics XM (Qualtrics) to obtain consent, collect demographic information, and administer patient-reported outcome measures (PROMs). PROMs allow us to collect quantitative data on subjective psychosocial constructs [21]. Participants were given the link to the Qualtrics survey and asked to complete the survey within 7 days; an interview was then scheduled. We conducted semistructured interviews via teleconference using Zoom (Zoom Video Communications) to collect qualitative data on participants’ experiences with COVID-19 restrictions. Prior to being used with study participants, the survey was pilot-tested with research team members and one participant who had experienced a stroke. In order to increase accessibility to the survey, if the person was unable to complete the survey online, it was administered over the telephone.

#### Demographic Information

Prior to the first interview, we collected data on participants’ age, city of residence, country of birth, sex, gender, first language, living situation, educational level, income at baseline, employment status, chronic illnesses, and status regarding COVID-19 (ie, whether they have tested positive or been exposed to someone who had tested positive).

#### Outcome Measures

##### Overview

The measures listed below were captured in the survey, which was to be completed 1 week prior to the interview at each time point. The measures are described in detail in Table 2, which documents the constructs measured, number of items, number of subscales, response ranges and anchors, and scoring.

**Table 2:** Outcome measures used to collect data at each of the four time points.

Measure	Construct	Items, n	Subscales, n	Names of subscales	Response range and anchors	Scoring and score range
Connor-Davidson Resilience Scale 25	Resilience	25	1	N/A^a^	0 (not true at all) to 4 (true nearly all the time)	0 (minimum) to 100 (maximum)
Hospital Anxiety and Depression Scale	Generalized anxiety and depression	14	2	Anxiety Depression	0 (none) to 4 (extreme)	0 (none/low) to 40 (extreme)
Keele Assessment of Participation	Activity participation	11	1	N/A	1 (all the time) to 5 (none of the time)	Restricted (some, little, none) and not restricted (all, most)
Life Space Assessment	Space mobility	9	1	N/A	Life-space level (1-5), the degree of independence (2=no assistance, 1.5=equipment only, and 1=personal assistance), and the frequency of movement (1=less than once a week, 2=1-3 times each week, 3=4-6 times each week, and 4=daily)	0 (totally bedbound) to 120 (moved out of town every day without assistance)
Multidimensional Scale of Perceived Social Support	Social support	12	3	Family Friends Significant other	1 (very strongly disagree) to 7 (very strongly agree)	12 to 84
Multidimensional State Boredom Scale	State boredom	29	5	Targeting disengagement High arousal Inattention Low arousal Time perception	1 (strongly disagree) to 7 (strongly agree)	29 to 203
Social Networking Usage Questionnaire	Social networking usage	19	1	N/A	1 (never) to 5 (always)	19 to 95
Technology Readiness Index 2.0	Individual’s technology readiness	16	4	Optimism Innovativeness Discomfort Insecurity	1 (strongly disagree) to 5 (strongly agree)	16 to 80

^a^N/A: not applicable; this subscale did not have a unique name.

##### Anxiety and Depression

The Hospital Anxiety and Depression Scale [22] measures anxiety and depression. Evaluation of this scale among primary care patients and the general public has demonstrated good concordance with clinical diagnoses of anxiety and depression, good sensitivity to change, and a mean Cronbach α of .83 [23].

##### Resilience

The Connor-Davidson Resilience Scale 25 [24] is a self-administered scale that measures resilience and how well people can cope with, and bounce back after, stressful events and tragedies. The reported Cronbach α for this measure is .93 [24] when used in a sample of adults from the general population.

##### Boredom

Boredom was assessed with the Multidimensional State Boredom Scale [25]. The Cronbach α for the total measure was .96 [26] upon development of the questionnaire.

##### Social Support

The Multidimensional Scale of Perceived Social Support [27] was used to assess perceived social support in three factor groups: family, friends, or significant other. The Cronbach α for the total score of this scale was .88 [27] in a sample including adolescents and adults.

##### Activity Participation

The Keele Assessment of Participation [28] measures participation in activities such as work, education, and socialization as well as in activities of daily living. The Cronbach α of this measure has been reported as .93 with a sample of adults aged 50 years and older [28].

##### Space Mobility

The Life Space Assessment reports on how frequently and far people have traveled out of the room in which they sleep during the previous 4 weeks. This assessment is moderately correlated with the Reintegration to Normal Living Index with Spearman ρ correlations ranging from 0.509 to 0.538. The 9-day test-retest reliability (intraclass correlation coefficient) has been reported to be 0.876 [29] among people with SCI.

##### Social Networking

The Social Networking Usage Questionnaire measures social media usage—academic, entertainment, and socialization—in online spaces. Questions regarding academic usage were removed from the questionnaire as these did not apply to our participants. A Cronbach α of .83 has been reported with university students [30].

##### Technology Use

The Technology Readiness Index 2.0 [31] is used to assess participants’ readiness to embrace new technologies. The Cronbach coefficients for all the dimensions were higher than .60 [32] in a heterogenous sample of adults aged 18 years and older.

##### Substance Use

The demographic questionnaire included five questions related to substance use; for example, “Do you use tobacco products?” and a clarification question asking, “Are you using more or less than you did before COVID-19?” These questions were repeated for marijuana use, alcohol, other drugs, and prescription drugs.

#### Semistructured Interviews

Following the completion of the surveys, participants took part in semistructured interviews on the University of British Columbia’s (UBC’s) secure Zoom platform to maintain physical distance. Three interview guides were created (Multimedia Appendix 1): a guide for the first interview at T1, a guide for the interviews at T2 and T3, and a guide for the final interview at T4. Participants were asked about their experiences with daily activities, changes in activity, their feelings about these changes, and strategies they used to cope with the impact of COVID-19 restrictions. Each interview guide was tested in three pilot interviews prior to being used with participants. Each participant was assigned the same interviewer for each of their interviews to facilitate rapport and relevance of follow-up questions based on previous interviews. At the start of the first interview, the interviewer confirmed any changes to the demographic information collected by the survey at each subsequent interview (eg, living arrangements and employment status).

Participants were also invited to take pictures during the course of the study that represent their daily and social experiences during the period of COVID-19 restrictions. The photos acted as a catalyst to enrich the sharing of information, as interviewers prompted participants to share the meaning behind the photos. Participants were invited to send photos to the research coordinator at each time point, which were then combined into a collage by the research team. The interviewer shared the collage with participants in the final interview and asked them to choose two to three photos from the collage and describe what the photos meant to them in the context of the COVID-19 restrictions.

The interviews were recorded on a password-protected Zoom account as well as on a voice recorder as a backup, to ensure accuracy of the transcription, and were then transcribed verbatim. Participants were anonymized with a unique participant ID; the key matching the ID with the name and contact information for each participant is kept in a password-protected and encrypted Microsoft Excel file on a secure UBC server. When transcribing the interviews, each participant’s ID was used to refer to them and other proper nouns were replaced with pseudonyms. Participants received a Can $30 (US $23.78) honorarium after each interview.

### Training

The data collection team consists of eight interviewers and 17 transcribers. Interviewers were trained three times over the course of the project: twice before data collection and once before the fourth and final interview. The latter session served as a refresher and provided training on new content added to the interview guide. Transcribers used transcription templates for all interviews and they time-stamped passages to be clarified or verified by the interviewer. Most members of the transcription team were experienced and did not require additional training. Novice transcribers received a tutorial from a study team member.

### Research Team Characteristics

The research team for this study is a large group of coinvestigators who are professors, educators, researchers, and other university employees. Interviewers and transcribers included volunteers who are university educated. Information on the positioning of the research team will be reported in further detail as relevant to each publication on the study findings.

### Data Analysis

#### Quantitative Data Analyses

Before conducting the quantitative analyses, data will be imported from Qualtrics to SPSS (IBM Corp). First, univariate statistics will be used to account for the number and percent of missing data in each measure. The patterns of missing values for each measure will then be visualized. Missing value patterns will be evaluated carefully to determine missing data mechanisms (ie, missing completely at random, at random, or not at random) [33]. In addition, to determine whether the observed missing value patterns are related to other variables in the study, for each measure that contains missing values, an indicator variable will be developed. The indicator variable will separate the participants who provided complete responses to that measure from the participants who did not complete that measure. Logistic regression will be used to determine whether other variables in the study, including demographic variables, can statistically predict the indicator variable. If none of the variables in the logistic regression analyses predicts the indicator variable, it can be concluded that the missing pattern mechanisms are missing completely at random or missing at random. If the values are missing completely at random or missing at random, and the percentage of the missing values is less than 30%, we will impute the missing values using a multiple imputation technique. Multiple imputation will result in multiple sets of plausible values for each missing value. In these analyses, the multiple imputation will be used to compute five plausible values for each missing value. The missing values will then be replaced by the mean of the five plausible values. Multiple imputation analyses will be run for each measure that contains missing values in each group separately. After imputing the missing values, to test our hypotheses and research questions, the following statistical analyses will be used. First, univariate analyses (eg, mean, sum, standard deviation, variance, range, and frequency) will describe the sample. In cross-sectional analyses, multivariate analyses of variance will be used to test whether there is a statistically significant difference between groups on the outcome variables. Correlational analyses, including the Pearson correlation coefficient (*r*), will be used to test the strength of the associations between different variables. Regression analyses (eg, linear regression and logistic regression) will be used to estimate the relationship between dependent variables and the outcome variables.

To analyze the longitudinal data, hierarchical linear modeling (HLM) growth curve analysis will be used to investigate the changes in the participants over time [34]. HLM growth curve analysis has several benefits over repeated-measures analysis of variance. First, HLM can be used when the interval between the time points is not equal and when data contain missing values. In addition, in contrast to the repeated-measures analysis of variance, HLM focuses on individual differences over time by considering each participant’s initial intercept and slope score, while repeated-measures analysis of variance focuses on the group differences [35].

#### Qualitative Data Analyses

Transcripts will be analyzed using content analysis to develop a qualitative description [18] of events and experiences of participants within and across the four time points. Analyses will be conducted separately for each of the participant groups—families, people with SCI, people with stroke, people with disabilities, and older adults—and each time point; they will then be combined, if appropriate, to examine trends over time or themes across groups [36]. A subteam of researchers will analyze each group. Because of the large research team, the blend of novice and experienced researchers, and the five participant groups, a consistent approach to coding will be facilitated by developing coding manuals with notations to explain coding decisions [37]. We anticipate creating multiple codebooks based on participant status, data collection time point, and omnibus analyses. These coding guides remain flexible to the possibility of adding new codes and recoding previous interviews. For each coding team, two primary coders will read and reread transcripts, code the first two to three interviews, compare codes, and discuss any discrepancies with the coding team. This coding guide will then be applied to subsequent interviews and revised in consultation with the qualitative research team for each participant group. During initial coding, the primary analysts will assign tentative codes to each idea reflective in the text, which are recorded in a codebook. The codebook and sample quotes supporting the codes will be shared with senior researchers. After integrating the comments from the senior researchers, the coders will code the next three to four interviews separately and once again compare their codes to ensure consistency in applying the codebook, updating as needed to reflect new or revised codes. The codebook developed at T1 will be used to code T2 interviews, updated with new codes developed at T2, which will be applied and updated at T3 and then again at T4; that is, codebooks will be updated as interviews are conducted throughout the longitudinal study. The coding procedure will be applied to each participant group separately, requiring four iterations of five participant group codebooks. Codes will then be organized into categories representing and interpreting the main topics shared by participants. The final qualitative description is expected to be presented as a set of themes and their interrelationships, supported by illustrative quotes from participants to explain their experiences of pandemic restrictions during the study period.

To integrate quantitative and qualitative data, there are three potential models of integration that may be used. Our primary approach will be to analyze both data sources separately. We will also explore the possibility of sequential analysis in which quantitative or qualitative analysis is used to inform a subsequent analysis using the alternative type of data [37] (eg, we may identify different types of experiences based on the qualitative data and compare scores on quantitative measures among participants who have similar experiences, or vice versa).

With regard to trustworthiness [38], an audit trail includes reflexive memos to document research discussions, possible biases, and analytical decisions (ie, dependability and confirmability). Comparing qualitative themes with numerical results from the PROMs will be used as a form of data triangulation and will generate questions to consider in refining qualitative themes. Researcher triangulation will occur within each subteam, in their analysis of a specific participant group, and in the larger team, where all researchers will be involved across all participant groups. Where available, member checking will further enhance analytical rigor.

## Results

Approval for the study was obtained from the UBC Behavioural Research Ethics Board (Approval No. H20-01109) on April 17, 2020. This research was unfunded. A total of 81 participants were enrolled in this study and data are being analyzed. Data analyses are expected to be completed in fall 2021; submission of multiple papers for publication is expected by winter 2021.

## Discussion

### Overview

Interviewing and surveying a heterogenous sample of participants during the COVID-19 restrictions present an opportunity for insight into perceived constraints, barriers, and strategies to cope with this unusual period. Therefore, our longitudinal investigation into changes in activity, participation, and well-being informs recommendations for the post–COVID-19 period and for future public health emergencies. We anticipate that this study will provide information to practitioners working in public health, with patient groups highlighted in this research, by identifying gaps in services. Further, the findings are relevant across disciplines, as mental health is increasingly a priority for health, education, and social service sectors, as well as government portfolios. With a better understanding of changes in physical and social activities and self-management strategies, there is potential for development of a guide that will provide information, resources, and strategies for managing periods of isolation. Future research in this area is needed to inform the development and evaluation of such a resource guide.

Our knowledge translation plan will target families with school-aged children, people with disabilities such as SCI and stroke, and older adults. We will leverage existing communication tools, such as websites (eg, health authorities in BC), presentations at provincial practice forums, and social media (eg, Twitter). Building on existing partnerships with the Canadian Association of Occupational Therapists in BC and our research centers (eg, GF Strong and ICORD [International Collaboration on Repair Discoveries]), a summary will be prepared for their websites and electronic newsletters to assist us with the implementation of our findings.

### Limitations

This study has two main limitations. First, this study relied on online or phone use for data collection; therefore, all participants needed access to and familiarity with the required technology. Findings are, therefore, generalizable to others with similar characteristics and advantages. Second, funding limitations restricted the number of participants we could recruit for each group, which may make some quantitative subgroup analyses challenging and limit our ability to achieve theoretical sufficiency with our qualitative analysis.

## Appendix

Multimedia Appendix 1 Interview guides for the interviewers.

## Abbreviations

BC: British Columbia
GRAMMS: Good Reporting of A Mixed Methods Study
HLM: hierarchical linear modeling
ICORD: International Collaboration on Repair Discoveries
PROM: patient-reported outcome measure
SCI: spinal cord injury
T1: time point 1
T2: time point 2
T3: time point 3
T4: time point 4
UBC: University of British Columbia
