# Supplementary material for: The Impact of COVID-19–Related Restrictions on Social and Daily Activities of Parents, People With Disabilities, and Older Adults: Protocol for a Longitudinal, Mixed Methods Study
Source: JMIR Res Protoc. 2021 Sep 1;10(9):e28337. doi: 10.2196/28337 (PMC8412136; doi:10.2196/28337)
Supplement: Multimedia Appendix 1 [file resprot_v10i9e28337_app1.docx]

Appendix 1: Sample interview guide

**Interview guide**

*Hello, my name is _____________ and I would like to start the interview by thanking you for your participation. It is greatly appreciated. The purpose of this interview is to explore and gain a deeper understanding of your experiences and perceptions during the COVID-19 pandemic.*

*Throughout the interview, you will be asked about your personal experiences. The interview will take approximately 60 minutes. Per the Informed Consent you signed, I just want to remind you that your participation is voluntary. You can stop the interview at any time, if you no longer want to participate. You also do not need to answer any questions which make you feel uncomfortable. During the interview, my main job is to listen to you and your stories. I look forward to hearing about your experiences.*

*As indicated in the Informed Consent, this session will be video recorded and then transcribed. I will let you know when I will start recording. If your name happens to come up in the interview, it will be removed when the interview is transcribed and replaced with a pseudonym. Do you have any questions for me before we start? I am now going to start recording.*

1. Tell me what you’ve learned over the past month during COVID.
2. What has your experience of COVID been like?

Prompt: Emotionally, physically (exercise), socially.

Prompt: Who, if anyone, do you discuss how you feel about COVID with?

1. What was a typical day like for you before the COVID quarantine?

Prompt: What does a typical day look like for you now?

Prompt: What is the most meaningful part of your day?

Prompt: Which of these changes do you feel are positive?

Prompt: Which of the changes do you feel are negative?

1. What are you doing more of?
2. What are you doing less of?
3. What activities are you doing to connect with others?
   Prompt: How would you describe your living situation?
4. What activities are you doing to contribute?
   Prompt: To your personal well-being, to society, to the world?
5. What type of restorative activities are you engaging in?
   Prompt: How do you feel when you are doing your daily activities?
6. How, if at all, has the meaning of the activities you do changes with the advent of COVID?
7. What are some of the biggest challenges you have encountered?
8. What are some of the strategies and supports you have used to overcome them?
9. Knowing the ways you’ve adjusted to the current situation, what would you recommend to others?
10. What ideas do you have about innovations that could facilitate that change?
    Prompt 1. How, if it all, has your use of social media changed during this time?
    Prompt 2: Describe your use of technology currently.
11. What are your future plans?

Prompt: Today, this week, monthly, before next interview, for the year

1. You will have the option to participate in a “photovoice” part of this study where you take photos of your experiences during quarantine and share them during the next interview, or send them to us online. You will also have the option of giving the research team consent for these photos to be used beyond the interview in the report and in subsequent publications and presentations. Taking and sharing photos is entirely voluntary, and the photos will only be used as a part of this project. Photos will be stored on an encrypted server. Please let us know whether or not you wish to participate in the photovoice in the survey we send to you before our next interview and sign the Photo Release Form if you choose to send and share photos with the research team.

**Subsequent interviews**

*Hi, again! I would like to start the interview by thanking you for your continuing participation. It is greatly appreciated.*

*Much like our last interview, you will be asked about your personal experiences. The interview will take approximately 60 minutes. Per the Informed Consent you signed, I just want to remind you that your participation is voluntary. You can stop the interview at any time, if you no longer want to participate. You also do not need to answer any questions which make you feel uncomfortable. During the interview, my main job is to listen to you and your stories. I look forward to hearing about how things have gone since we met!*

*As indicated in the Informed Consent, this session will be video recorded and then transcribed. I will let you know when I will start recording. If your name happens to come up in the interview, it will be removed when the interview is transcribed and replaced with a pseudonym. Do you have any questions for me before we start? I am now going to start recording.*

1. Since the last time we talked how have things been going for you?
2. Have you experienced any changes of (e.g., living situation, health, etc.)
3. What is a typical day like for you?
4. How, if at all, has your experience of COVID changed since we last talked?
5. How, if at all, has COVID affected what a typical day is like for you?
6. What activities are you doing to connect with others?
   Prompt: How would you describe your living situation?
7. What activities are you doing to contribute?
8. What type of restorative activities are you engaging in?
   Prompt: How do you feel when you are doing your daily activities?
9. How, if at all, has the meaning of the activities you do changes with the advent of COVID?
10. What are some of the biggest challenges you have encountered?
11. What are some of the strategies and supports you have used to overcome them?
12. What are some potential things that you would think would be helpful for people who are in similar situations as you?
13. What do you think should happen in that regard?
14. What ideas do you have about innovations that could facilitate that change?
    Prompt 1. How, if it all, has your use of social media changed during this time?
    Prompt 2: Describe your use of technology currently?
15. (If consented to photovoice) Please describe each picture you took
    Prompt: Why did you take this picture? What does it convey about your experience?
16. What are your future plans?

**Interview 4 guide**

*Hi, again! I would like to start the interview by thanking you for your continuing participation. It is greatly appreciated.*

*Much like our last interview, you will be asked about your personal experiences. The interview will take approximately 60 minutes. Per the Informed Consent you signed, I just want to remind you that your participation is voluntary. You can stop the interview at any time, if you no longer want to participate. You also do not need to answer any questions which make you feel uncomfortable. During the interview, my main job is to listen to you and your stories. I look forward to hearing about how things have gone since we met!*

*As indicated in the Informed Consent, this session will be video recorded and then transcribed. I will let you know when I will start recording. If your name happens to come up in the interview, it will be removed when the interview is transcribed and replaced with a pseudonym. Do you have any questions for me before we start? I am now going to start recording.*

**Photovoice**: (if provided) open provided slideshow and share with participant. Skip to question 7 if participant has no photos

**Note** for photovoice: You may make your own comments as well. Perhaps even just describing it visually could get things going.

You could also ask some leading questions if participants aren’t digging deep themselves into the content.

Try to be open and not shut people down. Curiosity—learn as you go.

1. Over the several times we interviewed you, you provided pictures. I want to share with you a collage of your pictures. From these pictures I would like to identify a couple that speak about your COVID experience.
2. Tell me about this picture. What does it mean to you? What was going through your mind when you took the picture?

Prompt: Based on the pictures you identified, can you tell me when you took them and what period of time they represented

1. How have things in your life changed for you since you uploaded this picture?

Probe: looking at this picture NOW, how does it make you feel?

1. What do you think you’re learning as you re-visit these pictures?
2. Since the last time we talked how have things been going for you?
3. What changes have you experienced since we last talked:
   Prompt: living situation, health, etc.

Prompt: [follow up on notes from last interview, e.g. “last time you also mentioned ...”]

Prompt: what has remained the same?

1. What is a typical day like for you right now?

Prompt: What is the most meaningful part of your day?

Prompt: What are you doing more of?

Prompt: What are you doing less of?

1. How, if at all, has your experience of COVID changed since we last talked?

Prompt: What, if any, discrimination have you experienced that you think might be related to COVID?

1. How, if at all, has COVID affected what a typical day is like for you?
2. What activities are you doing to connect with others?
   Prompt: How would you describe your living situation since we last talked?
3. What activities are you doing to contribute since we last talked/currently?

Prompt: To your personal well-being, to society, to the world?

1. What type of restorative activities are you engaging in since our last interview?
   Prompt: How do you feel when you are doing your daily activities?
2. Is there something that you think is unique about your life that has influenced your COVID experience?

Prompt: If you identify as an individual with a disability, how do you think your disability has influenced your COVID experience?

1. How, if at all, has the meaning of the activities you do changes with the advent of COVID since we last talked?
2. What are some of the biggest challenges you have encountered since our last interview?
3. What are some of the strategies and supports you have used to overcome them?
4. Have you been admitted to the hospital since the beginning of the COVID pandemic in March 2020?

Prompt: If yes, do you mind telling me what your experience was like?

Prompt: Do you mind telling me why you were admitted?

1. How do you feel about the COVID vaccine? Will you be getting one?
2. Having experienced a second wave of the COVID pandemic what suggestions would you provide to help people navigate through this time?

Prompt: How have you dealt with this lockdown compared to the first time?

Prompt: Did you prepare at all for another phase of isolation after the first wave?

Prompt: What did you do to prepare?

1. What, if any, innovations or new technologies are you using to help get by during this pandemic (e.g., for sports, recreation, socializing, mobility)?

Prompt: What do you like/dislike about these technologies.

Prompt: What ideas do you have about innovations that could facilitate that change?
Prompt: How, if it all, has your use of social media changed since we last talked?
Prompt: Describe your use of technology currently?

1. What are your future plans?

Prompt: Today, this week, monthly, before next interview, for the year. *closing thoughts are very interesting so be sure to continue recording. If it is about time to end the interview, let them know and thank them again a final time.*
